# Supplementary material for: HIV testing uptake and yield among sexual partners of HIV-positive men who have sex with men in Zhejiang Province, China, 2014-2016: A cross-sectional pilot study of a choice-based partner tracing and testing package
Source: PLoS One. 2020 Jun 4;15(6):e0232268. doi: 10.1371/journal.pone.0232268 (PMC7272034; doi:10.1371/journal.pone.0232268)
Supplement: S1 Fig — (PDF) [file pone.0232268.s001.pdf]

- **Couples' HIV counseling and testing (CHCT):** Based on dual referral, in which a trained provider accompanies and provides support to HIV-positive patients when they disclose their status and the potential exposure to HIV infection to their partners.[17] The IC is requested to return to the VCT clinic with his sexual partner at a subsequent date to receive joint HIV testing and counseling. The IC is not requested to disclose his HIV status to his sexual partner prior to the CHCT session. The VCT staff follow up with a phone call to the IC once monthly until the IC and his sexual partner complete the CHCT session during the defined follow-up period of three months.

- **Information assisted partner notification (IAPN):** Based on provider referral, in which a trained provider confidentially contacts the patient's partner(s) directly and offers the partner(s) voluntary HTS. The IC is requested to provide social media and phone contact information, such as accounts of social media or dating, phone number and so on.[17] The VCT staff then contact the sexual partner through these communication methods. The VCT staff declares his/her own identity and explains that his/her information was provided by one of his/her sexual partner; communicate his/her risk of HIV infection; promote VCT; provide information of local VCT clinics. The VCT staff does not reveal the IC's identity (such as name, telephone number, social account or other personal information). The VCT staff follows up with a communication to the sexual partner once monthly until s/he attends a VCT for HTC during the defined follow-up period of three months.

- **HIVST:** Based on Directly assisted HIV self-testing (HIVST), in which individuals who are self-testing for HIV receive an in-person demonstration from a trained provider or peer before or during HIVST.[17] The IC is requested to undergo an information session on oral rapid HIV testing kit by the VCT staff, receive a kit, provide the kit for and assist in testing his sexual partner, and return the testing kit itself by ICs to the VCT clinic within two weeks. If the returned testing kit indicates a positive result, the VCT staff request that the IC either (a) request that his sexual partner comes to the VCT clinic for a confirmatory test; or (b) provide social media and phone contact information for his sexual partner so that the VCT staff may initiate tracing. The VCT staff follow up with a phone call to the IC once monthly until his sexual partner attends HIV testing during the defined follow-up period of three months.

- **Patient referral:** The ICs is requested to notify his sexual partner of his positive status and suggest HTS.[17] The ICs provide social-demographics of sexual partners to confirm his/her identity during the following HIV testing. The VCT staff follow up with a phone call to the IC once monthly until his sexual partner attends a VCT for HIV testing during the defined follow-up period of three months.

Figure1 Introduction for modes in the partner tracing and HIV testing package among sexual partners of newly diagnosed HIV positive men who have sex with men, Zhejiang Province, China
