# Supplementary figures and images for: HIV testing uptake and yield among sexual partners of HIV-positive men who have sex with men in Zhejiang Province, China, 2014-2016: A cross-sectional pilot study of a choice-based partner tracing and testing package
Source: PLoS One. 2020 Jun 4;15(6):e0232268. doi: 10.1371/journal.pone.0232268 (PMC7272034; doi:10.1371/journal.pone.0232268)

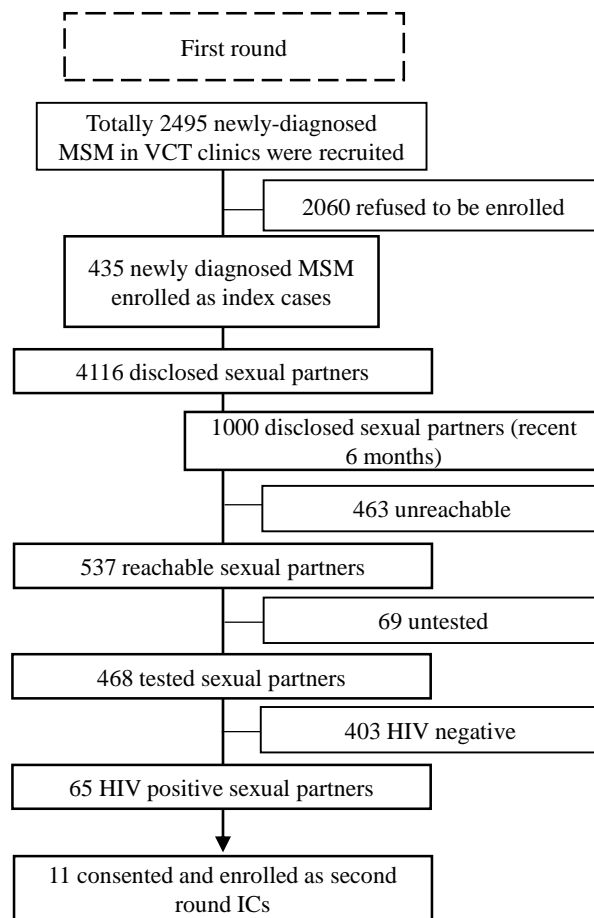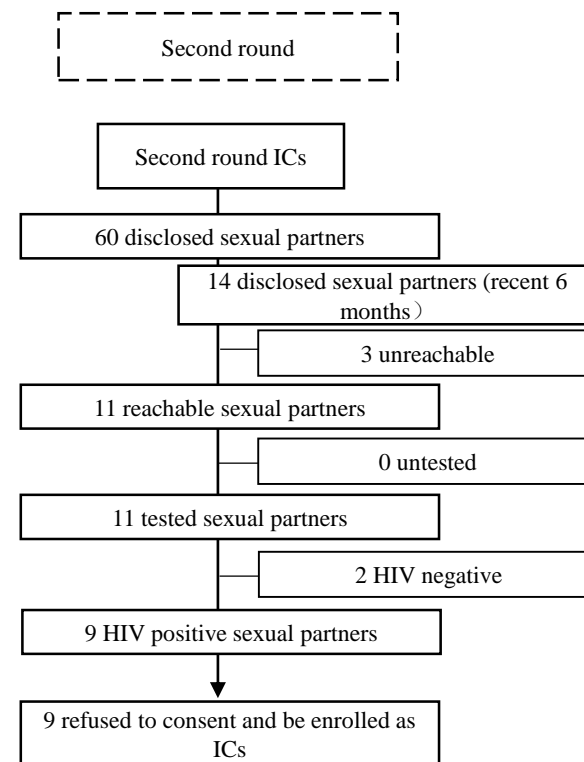

Supplement: S2 Fig — (PDF) [file pone.0232268.s002.pdf]
