# Supplementary material for: HIV testing uptake and yield among sexual partners of HIV-positive men who have sex with men in Zhejiang Province, China, 2014-2016: A cross-sectional pilot study of a choice-based partner tracing and testing package
Source: PLoS One. 2020 Jun 4;15(6):e0232268. doi: 10.1371/journal.pone.0232268 (PMC7272034; doi:10.1371/journal.pone.0232268)
Supplement: S1 Table — (PDF) [file pone.0232268.s003.pdf]

Table 4: HIV testing uptake by modes in different types of sexual relationship among reachable sexual partners of newly diagnosed HIV positive MSM in June 2014 through June 2016, in Hangzhou and Ningbo Cities, China.

Type(s) of sexual relationship with sexual partners	Modes of partner tracing and testing package	Tested	Not tested	χ^2	*p*-value
Stable, non-commercial same-sex relationship/ opposite-sex spouse	CHCT	120(88.2)	16(11.8)	2.827	0.419
	IAPN	56(94.9)	3(5.1)		
	HIVST	10(90.9)	1(9.1)		
	Patient Referral	80(93.0)	6(7.0)		
Casual, non-commercial same-sex relationship	CHCT	39(68.4)	18(31.6)	-	<0.001

Type(s) of sexual relationship with sexual partners	Modes of partner tracing and testing package	Tested	Not tested	χ^2	*p*-value
	IAPN	89(93.7)	6(6.3)		
	HIVST	5(100.0)	0(0.0)		
	Patient Referral	67(87.0)	10(13.0)		
Commercial same-sex relationship / Unmarried opposite-sex relationship/ Others	CHCT	3(75.0)	1(33.3)	-	0.392
	IAPN	1(100.0)	0(0.0)		
	HIVST	-	-		
	Patient Referral	6(75.0)	2(25.0)		

OR: Odds Ratio; CI: Confidence Interval; Neg: negative HIV test; Pos: positive HIV test; CHCT: couples' HIV testing and counseling; IAPN: Information assisted partner notification; HIVST: assisted HIV self-testing
