# Supplementary material for: HIV testing uptake and yield among sexual partners of HIV-positive men who have sex with men in Zhejiang Province, China, 2014-2016: A cross-sectional pilot study of a choice-based partner tracing and testing package
Source: PLoS One. 2020 Jun 4;15(6):e0232268. doi: 10.1371/journal.pone.0232268 (PMC7272034; doi:10.1371/journal.pone.0232268)
Supplement: S1 File — (DOCX) [file pone.0232268.s004.docx]

Characteristics of HIV positive MSM, Zhejiang

{NO}NO. of questionnaire：ICID_______________

{NAME} Name：NAME________________ {ID}

Card ID of HIV/AIDS：ID______________________

{PHONE} Tel：PHONE_______________

{A01}Age of your first same sex contact：##（years old）

{A02}Your major role in anal sex behavior with same sexual contacts：#①Attack ②Receive ③Both

{A03} What are your sexual orientation? # {A03A} ①Homosexual ②Heterosexual ③Bisexuality ④Indeterminate ⑤Other：A03a________________(Please state)

B Sexual contacts

B1 Stable, non-commercial same-sex contact

{B1a} Total number of Stable, non-commercial same-sex contacts ###

{B2a} Number of Stable, non-commercial same-sex contacts in recent 6 months###

{B3a} Number of Stable, non-commercial same-sex contacts, still in a relationship###

{B4a} Number of Stable, non-commercial same-sex contacts, still in touch##

B2 Casual, non-commercial same-sex contact

{B1b} Total number of casual, non-commercial same-sex contacts ###

{B2b} Number of casual, non-commercial same-sex contacts in recent 6 months###

{B3b} Number of casual, non-commercial same-sex contacts, still in b relationship###

{B4b} Number of casual, non-commercial same-sex contacts, still in touch##

B3 Commercial same-sex contact (MB)

{B1c} Total number of commercial same-sex contacts ###

{B2c} Number of commercial same-sex contacts in recent 6 months###

{B3c} Number of commercial same-sex contacts, still in b relationship###

{B4c} Number of commercial same-sex contacts, still in touch##

B4 Commercial same-sex contact (Guest)

{B1d} Total number of commercial same-sex contacts ###

{B2d} Number of commercial same-sex contacts in recent 6 months###

{B3d} Number of commercial same-sex contacts, still in b relationship###

{B4d} Number of commercial same-sex contacts, still in touch##

B5 Opposite-sex spouse

{B1e} Total number of opposite-sex spouses ###

{B2e} Number of opposite-sex spouses in recent 6 months###

{B3e} Number of opposite-sex spouses, still in b relationship###

{B4e} Number of opposite-sex spouses, still in touch##

B6 Unmarried opposite-sex contact

{B1e} Total number of unmarried opposite-sex contacts ###

{B2e} Number of unmarried opposite-sex contacts in recent 6 months###

{B3e} Number of unmarried opposite-sex contacts, still in b relationship###

{B4e} Number of unmarried opposite-sex contacts, still in touch##

C Sex contacts in touch

Sex contacts G1：

{C1a}Type of sexual relationship： @# ① Stable, non-commercial same-sex contact ② Casual, non-commercial same-sex contact ③ Commercial same-sex contact（MB）④ Commercial same-sex contact（Guest）⑤ Opposite-sex spouse ⑥Unmarried opposite-sex contact ⑦Others：C1aa___________________ (Please state)

{C2a} Name or nickname：@____________

{C3a}Age（years old）： @## years old

{C4a} methods for searching for specific sexual cotact： @# {C4AA} ① Bar ② Public bathroom ③ Park/ Public latrine ④ Internet ⑤ Others：C4aa____________________

{C5a} Contact information（Telephone number; Accounts of QQ、WeChat and others）：@_______________

{C6a} Current relationship status @# ① Currently in a relationship ② No current relationship ③ Not sure

{C7a} Frequency of sexual contact, stable partners/spouse, as reported by index case(times/per week) in recent 6 months： @# ①<1 ②1-2 ③3-4 ④5-6 ⑤≥7

{C8a} Frequency of sexual contact, casual partners (total times) in recent 6 months：@###

{C9a}Condom use between sexual contact and IC： @# ①Never ② Inconsistent ③ Consistent

{C10a} Intervention modes：@# {C10AA} ① Couples’ HIV testing and counselling ② Information-driven assisted partner notification ③assisted HIV self-testing④ Routine (Patient referral) ⑤ Others：C10aa____________________

{C11a}Date of intervention（e.g. 2000/01/01）： @<yyyy/mm/dd>

{C12a} Attitude of HIV positive MSM（1-3 jump to C13a）： @# ① Strongly agree ②Agree ③ Reluctantly agree ④ Disagree ⑤Strongly disagreee

{C121a}If HIV positive MSM choose “disagree”，Please state the reason（End the survey）：______________________________

{C13a}Sex contact accept HIV testing or not(“1” jump to C14）：@# 0-No，1-Yes

{C131a}State the reason why sex contact reject HIV testing（End the survey）： @______________________________

{C14a}Date of sexual contact accept HIV testing（e.g. 2000/01/01）： @<yyyy/mm/dd>

{C15a}Results of syphilis testing： @# {C15AA} 0-Negative，1-ELISA/TPPA positive，2-RPR/TRUST positive，3-Both positive，4-Others：C15aa____________

{C16a}Results of HIV testing： @# {C16AA} 0-Negative， 1-Positive (Confirmed)， 2-Positive (Screening)，3-Others：C16aa____________

{C17a}If HIV positive，Card ID of HIV/AIDS：@____________________

{G1} End the survey or not（“1” End）：# 0-No，1-Yes

{D1} Survey Sites：D1__________________ {D2} Researcher：D2__________ Date（e.g.：2000/01/01）：{D3} <yyyy/mm/dd>
