# Supplementary material for: HIV testing uptake and yield among sexual partners of HIV-positive men who have sex with men in Zhejiang Province, China, 2014-2016: A cross-sectional pilot study of a choice-based partner tracing and testing package
Source: PLoS One. 2020 Jun 4;15(6):e0232268. doi: 10.1371/journal.pone.0232268 (PMC7272034; doi:10.1371/journal.pone.0232268)
Supplement: S2 File — (DOCX) [file pone.0232268.s005.docx]

**浙江省MSM阳性者信息收集表**

{NO}问卷编号：ICID_______________

{NAME} 姓名：NAME________________ {ID} 卡片编号：ID______________________ {PHONE} 联系方式：PHONE：_______________

{A01}您首次同性性行为的年龄：##（周岁）

{A02}您与同性发生肛交性行为时所扮演的角色：#①1号为主②0号为主③两者都有

{A03}您认为自己是:# {A03A} ①同性恋 ②异性恋 ③双性恋 ④未确定 ⑤其他：A03a________________(请注明)

B 性伴情况

B1 同性固定性伴

{B1a} 到目前为止总人数 ###

{B2a} 最近6个月人数 ###

{B3a} 仍保持性关系人数 ###

{B4a} 能联系上人数 ##

B2 同性临时性伴

{B1b} 到目前为止总人数 ###

{B2b} 最近6个月人数 ###

{B3b} 仍保持性关系人数 ###

{B4b} 能联系上人数 ##

B3 同性商业性伴（MB）

{B1c} 到目前为止总人数 ###

{B2c} 最近6个月人数 ###

{B3c} 仍保持性关系人数 ###

{B4c} 能联系上人数 ##

B4 同性商业性伴（客人）

{B1d} 到目前为止总人数 ###

{B2d} 最近6个月人数 ###

{B3d} 仍保持性关系人数 ###

{B4d} 能联系上人数 ##

B5 配偶

{B1e} 到目前为止总人数 ###

{B2e} 最近6个月人数 ###

{B3e} 仍保持性关系人数 ###

{B4e} 能联系上人数 ##

B6 其他异性性伴

{B1f} 到目前为止总人数 ###

{B2f} 最近6个月人数 ###

{B3f} 仍保持性关系人数 ###

{B4f} 能联系上人数 ##

C介绍可以联系上的性伴

性伴G1：

{C1a}性伴类型： @# ①同性固定性伴②同性临时性伴③同性商业性伴（MB）④同性商业性伴（客人）⑤配偶 ⑥其他异性性伴 ⑦其他：C1aa____________________

{C2a}姓名或昵称（与附录7对应）： @____________

{C3a}年龄（周岁）： @##岁

{C4a}结识途径： @# {C4AA} ①酒吧 ②浴池 ③公园/公厕 ④网络 ⑤其他：C4aa____________________

{C5a}联系方式（手机号、QQ、微信等）：@_______________

{C6a}目前性关系维持 @# ①继续 ②已经结束 ③不知道

{C7a}最近6个月性行为频率（仅固定性伴、配偶填）： @# ①小于1次/周②1-2次/周③3-4次/周④5-6次/周⑤大于7次/周

{C8a}最近6个月性行为次数（除固定性伴、配偶外者填）：@###次

{C9a}安全套使用： @# ①从不使用②有时使用③每次都用

{C10a}性伴检测驱动方式：@# {C10AA} ①伴侣咨询检测 ②信息导向驱动检测 ③唾液自助检测 ④常规推动方式（性伴自我告知） ⑤其他：C10aa____________________

{C11a}开始动员时间（格式：2000/01/01）： @<yyyy/mm/dd>

{C12a}MSM阳性者接受程度（1-3跳至C13）： @# ①非常同意②同意③勉强同意④不同意⑤坚决不同意

{C121a}如果MSM阳性者不愿意，请填写原因（结束调查）：______________________________

{C13a}是否成功取得性伴进行检测（1跳至C14）：@# 0-否，1-是

{C131a}性伴不检测的原因（结束调查）： @______________________________

{C14a}性伴检测时间（2000/01/01）： @<yyyy/mm/dd>

{C15a}梅毒检测结果： @# {C15AA} 0-均阴性，1-ELISA/TPPA阳性，2-RPR/TRUST阳性，3-均阳性，4-其他：C15aa____________

{C16a}HIV检测结果： @# {C16AA} 0-阴性， 1-确证试验阳性， 2-初筛阳性未确诊，3-其他：C16aa____________

{C17a}如果阳性，卡片编号：@____________________

{G1}是否跳转（“1”调查结束）：# 0-否，1-是

{D1} 调查单位：D1__________________ {D2} 调查员：D2__________ 调查时间（格式：2000/01/01）：{D3} <yyyy/mm/dd>
