# Supplementary material for: HIV testing uptake and yield among sexual partners of HIV-positive men who have sex with men in Zhejiang Province, China, 2014-2016: A cross-sectional pilot study of a choice-based partner tracing and testing package
Source: PLoS One. 2020 Jun 4;15(6):e0232268. doi: 10.1371/journal.pone.0232268 (PMC7272034; doi:10.1371/journal.pone.0232268)
Supplement: Attachment — Submitted filename: Review 1210.docx [file pone.0232268.s006.docx]

Thank you for the opportunity to review the manuscript titled “HIV testing uptake and yield among sexual contacts of HIV-positive men who have sex with men in Zhejian Province, China, 2014-2016: a cross-sectional pilot study of a choice-based partner tracing and testing package”. This study examined the uptake and yield of four different partner tracing mechanisms and provided recommendations for how to best improve contact tracing. This manuscript is well conceptualized and can be improved by addressing the following comments:

Methods

- Please explicitly state the time frame (e.g., past month, past 2 months, etc.) in which someone would be considered “newly diagnosed as HIV positive”.
- The sentence in lines 109-111 is a bit hard to understand.
- Were sexual contacts of IC who were under the age of 18 and who were HIV-negative also included in this study? You explicitly mentioned sexual contacts under 18 who were HIV-positive.
- Given the nature of this study, it may be important to include the IC’s viral load data in this analysis, since this metric is often used to estimate potential risk of forward transmission.
- What type of regression did you use? You presented odds ratios, which makes me assume logistic regressions were used.
- It may be worthwhile to examine if sexual contacts who are in different types of relationships prefer certain methods of contact. For example, if the sexual contact is in a relationship with someone of the opposite sex, the sexual contact may be hesitant to appear with his male sexual partner in public.
- Were there processes in place to make sure that sexual contacts were not duplicated? For example, if two different ICs identified the same sexual contact, this may affect the validity of the post estimates.
- It may be important to explain exactly why those with severe AIDS complications were excluded. If the authors considered high viral load/low cd4 count as a severe complication, those at highest risk for forward transmission may be excluded from this study.

Results

- The first three 95% Cis presented in lines 222-226 all cross the null, however, they are presented as if they were statistically significant.

Discussion

- You mention that only a small proportion of potential participants enrolled in this study. Would it be possible to compare those who enrolled to those who did not to identify if there are certain characteristics that differ between the two groups?

Minor:

Line 186 – Change 4176 to 4,176

Line 187 – please add a space between 548 and (13%)

Line 195 – please add a space between 31.5 and (SD=9.6)

Line 198 – please add a space between 30.7 and (SD=8.9)

It may be worthwhile for the authors to go through the manuscript to identify additional grammatical errors.
