# Supplementary material for: HIV testing uptake and yield among sexual partners of HIV-positive men who have sex with men in Zhejiang Province, China, 2014-2016: A cross-sectional pilot study of a choice-based partner tracing and testing package
Source: PLoS One. 2020 Jun 4;15(6):e0232268. doi: 10.1371/journal.pone.0232268 (PMC7272034; doi:10.1371/journal.pone.0232268)
Supplement: Attachment — Submitted filename: Response to Reviewers.docx [file pone.0232268.s007.docx]

**Response to Reviewers**

Dear editor and reviewers,

We appreciate it for your efforts to review this manuscript.

Thank you for the reviewers’ comments. We have revised the manuscript and especially paid attention to your comments and suggestions.

We have changed “sexual contact” to “sexual partner” in this revised manuscript as we think this description is much more accurate to define a person who had sex with IC.

In this revised version. Mr. Qiaoqin Ma has helped a lot with improving the description and related analysis. We authors have agreed that he should be listed as co-corresponding author.

Answers to reviewers’ comments are as follows:

Part 1:

| Reviewers’ comments | Response |
| --- | --- |
| Reviewer #1: |  |
| Congratulations on a fascinating project. This paper articulates the study design and the relevant findings. Also, the methods are sound. You could however explain a little more fully and in detail the different methods of partner tracing (i.e. not all readers will understand patient referral without an explanation).  My greatest concern with this paper is the written English. The paper needs to be revised by a native English speaker/writer which will allow these important findings to be understood more clearly and easily by the relevant audience. | Thanks for the reviewer’s comments. We have revised the method section, and provided details about different modes of partner tracing and HIV testing package.  About the written English, we have asked a native English speaker to help revise the manuscript and edit the language. We also have searched for language editing service from [www.editage.cn](http://www.editage.cn). |
| Reviewer #2: |  |
| 1. The authors conclude that the partner tracing and testing package was “effective for case-finding among a high-risk population.” However, it is unclear what criteria were used to determine “effectiveness”, as there were no outcomes measures for a comparison group that did not receive the intervention. Moreover, the authors’ optimistic appraisal does not seem very strongly supported by the data. The arguably low participation in the intervention (18%) and arguably low proportion of reachable contacts (12%), would lead many readers to conclude that the intervention was in fact not very effective. What if the participation rate and proportion of reachable contacts were only 5%? What was the cut-off point defining effectiveness, if any? Would the authors still conclude that the intervention was “effective”, as long as a relatively high proportion of reachable contacts of completed testing? Please provide stronger justification for why the intervention should be considered “effective”, or modify the appraisal regarding the effectiveness of the intervention. In addition, further discussion is needed about why the participation and reachable contact rates were so low, and what can be done to improve greater uptake. | Thanks for reviewer’s comments. We have seriously taken this comment into consideration. One important reason for overall low uptake might be that the number of self-disclosed sexual partners was the number of total sexual partners during one IC’s life. We consider that those reachable sexual partners may mostly come from those who are still in touch with ICs in recent period of time, thus we analyze the number of sexual partners in recent 6 months disclosed by index case, and to reflect the real gap between disclosed sexual partners and reachable sexual partners in the limitation part of discussion section. The participation rate of index cases varied among different types of HIV positive populations.  In this revised manuscript, we have used “feasible” in place of “effective” (lines 343-366). |
| 2. In the interest of implementation and replication, much more detail about the intervention (each mode) is needed. For example, for the “information-driven assisted partner notification” mode of intervention, how did staff introduce themselves when reaching out to contacts of the indexes? What was their training? How were they able to ensure confidentiality? Who covered the cost of testing of partners? For the “patient referral” intervention mode, how did study investigators ascertain the HIV test results of their contacts? | Many thanks.  We have revised the method section. All the details about introduction process, confidentiality, training, cost of testing, ascertaining testing results have been provided in the method section (lines 146-165), and we have updated the supplemental figure 1 to show this in detail.  All our co-authors have agreed that the English translation for “information-driven assisted partner notification” mode is redundant, and have decided to use “information assisted partner notification” to describe this mode. |
| 3. In the abstract and text, it appears that the authors did not meaningfully differentiate in their presentation of statistically significant and non-statistically significant results. For example, in the abstract: “odds of a reachable sexual contact enrolled in 28 information-driven mode taking an HIV test were 90% more than that of one enrolled in 29 patient referral (95%CI:0.8, 4.4).” Why conduct significance tests and state “We assigned statistical significance at p-values less than 0.05”, if the presentation of significant results are basically the same as non-significant results? | We have revised related section, changed reference group in this categorical variable (modes), and changed the description, in related sections (lines 28-30, 265-269) based on reviewer’s comments. |
| 4. Given the fact that partner contacts were clustered within indexes, I am concerned that the analytic approach did not control for correlated data. Please reanalyze the data using an analytic approach that controls for clustering of observations within indexes (e.g., GEE or random effects), or provide strong justification for why such an analysis is unnecessary. | Thanks, we have seriously considered this comment.  Now, we used univariate logistic regression to compare HIV testing uptake and yield of sexual contacts across different modes. In this study, we found that 18% (79/446) ICs have more than 1 reachable sexual partners, therefore we agree that potential correlation within clustered sexual partners should be considered in analysis process.  The estimated effect size now is produced and represented using odds ratios through generalized linear mixed models (GLMMs). In this model, whether or not sexual partners were clustered with the same IC, sexual partners’ age, and sexual partners’ gender are included to control potential correlation. We have revised related sections (lines 28-30, 205-211). |
| 5. Numerous grammatical errors need to be corrected. For example, “We defined reachable sexual contacts as those can be contacted…” (pg 7). | Thanks, we have revised the whole manuscript to correct these grammatical errors, and we have searched for language editing service from [www.editage.cn](http://www.editage.cn) to improve the description. |
| Reviewer #3: |  |
| This paper was well conceptualized, however, there are instances where the writing does not follow standard English grammatical conventions. It may be prudent of the authors to have a proof reader who can ensure that sentence structure and the usage of punctuation are sound. | Thanks for the reviewer’s comments. We have revised the written English through the whole manuscript. We also ask a native English speaker to help revise the manuscript, and we have searched for language editing service from [www.editage.cn](http://www.editage.cn) to improve the description. |

Part 2:

| Details | Response |
| --- | --- |
| Methods |  |
| • Please explicitly state the time frame (e.g., past month, past 2 months, etc.) in which someone would be considered “newly diagnosed as HIV positive”. | Thanks for the reviewers’ comments. Now we explicitly state the time frame as being diagnosed in one month in which someone would be considered “newly diagnosed as HIV positive”. We have revised the method section to make the description clearer, in lines 108, 137-138. |
| • The sentence in lines 109-111 is a bit hard to understand. | This sentence was to state the definition of “reachable sexual contacts”. We have revised this sentence to make the expression clearer. The revised version is “We defined reachable sexual contacts as those who could be contacted by face-to-face, official landline/telephone, social software (such as QQ, WeChat and Blued) or other communication tools.” In lines 117-119 now. |
| • Were sexual contacts of IC who were under the age of 18 and who were HIV-negative also included in this study? You explicitly mentioned sexual contacts under 18 who were HIV-positive. | Yes, there was one male sexual partner of IC, who was 17 years old, and HIV negative, was included in this study.  We provide standard post-testing counselling, and all participants tested negative would be exposed to health education and behavior intervention. We have made it clear in lines 173-175. |
| • Given the nature of this study, it may be important to include the IC’s viral load data in this analysis, since this metric is often used to estimate potential risk of forward transmission. | In China, ARV and viral load testing is provided to HIV positive patients free of charge by the government. We couldn’t collect viral load data of all newly diagnosed IC because viral load testing for free is not required to be done at the moment when a patient is newly diagnosed, just required to be provided once a year to the patients. |
| • What type of regression did you use? You presented odds ratios, which makes me assume logistic regressions were used. | Yes, we used univariate logistic regression to compare HIV testing uptake and yield of sexual partners across different modes.  In this revised manuscript, we have updated the analysis through generalized linear mixed models (GLMMs) to control potential correlation within clustered sexual partners (lines 205-211). |
| • It may be worthwhile to examine if sexual contacts who are in different types of relationships prefer certain methods of contact. For example, if the sexual contact is in a relationship with someone of the opposite sex, the sexual contact may be hesitant to appear with his male sexual partner in public. | Yes, we agree that sexual partners who are in different types of relationships prefer certain methods of contact. In this study, four modes for partner tracing and HIV testing were introduced to each IC, who then choose a mode which who think that this is the best and appropriate to him and his sexual partners.  On the other hand, unlike the western, two Chinese men may not hesitate to appear together in public because it won’t bring any suspicions that they are MSM or sex partners in Chinese culture as long as they don't act intimately even though one of them is in a relationship with someone of the opposite sex. |
| • Were there processes in place to make sure that sexual contacts were not duplicated? For example, if two different ICs identified the same sexual contact, this may affect the validity of the post estimates. | Thanks for your comment.  This study was conducted at 23 VCT clinics of 23 local CDCs in Hangzhou and Ningbo, 2 big cities in Zhejiang Province, China. Standardized VCT service is provided to each VCT seeker at VCT clinics, which is professional, confidential, free of charge and client friendly. Theoretically, one partner in this study is less likely to go to another VCT clinics again after he/she visited one clinic requested by the study staff. Further, we didn’t find duplicated sexual partners who is HIV negative using IC’s partners information such as social software accounts, phone numbers to match each other.  All HIV positive person must be reported to China national HIV/AIDS reporting system. Verification of duplicate case reporting is done when one case is reported. Thus, duplicate IC or IC’s HIV positive partner in this study could not be an issue to be noticed. |
| • It may be important to explain exactly why those with severe AIDS complications were excluded. If the authors considered high viral load/low cd4 count as a severe complication, those at highest risk for forward transmission may be excluded from this study. | In inclusion and exclusion section, we have revised this section into “severe AIDS complication or other illnesses requiring hospitalization”, in lines 112-113, to make it much clearer. |
| Results |  |
| • The first three 95% Cis presented in lines 222-226 all cross the null, however, they are presented as if they were statistically significant. | Based on the reviewers’ comment, we changed reference group in this categorical variable (modes), reanalyzed the dada and have made revision in related sections (lines 275-281). |
| Discussion |  |
| • You mention that only a small proportion of potential participants enrolled in this study. Would it be possible to compare those who enrolled to those who did not to identify if there are certain characteristics that differ between the two groups? | Thanks for your comment.  We couldn’t collect the characteristics about IC’s sexual partners who are not reachable. Therefore, we were not able to compare the differences between those IC who enrolled and those who did not.  We could just collect social-demographics for IC, such as age, education status, marital status and residence. We have compared those HIV positive MSM who enrolled to those who did not, and have found that those who settled in the detection place vs. those outside ( 28.5% vs. 12.3%) , those with education level of college or above vs. those with lower education level ( 20.5% vs. 16.0%) would be more likely to be enrolled to the program, and there was no statistical difference and there was no statistical difference for the variables of age and marital status. This comparison has also been stated in the discussion section as a limitation. (lines 353-358) |
| Minor |  |
| Line 186 – Change 4176 to 4,176  Line 187 – please add a space between 548 and (13%)  Line 195 – please add a space between 31.5 and (SD=9.6)  Line 198 – please add a space between 30.7 and (SD=8.9)  It may be worthwhile for the authors to go through the manuscript to identify additional grammatical errors. | Thanks for your advices, we have revised related sections and go through the manuscript to revise other grammatical errors. |

Part 3:

| Additional requirements | Response |
| --- | --- |
| 1. Please ensure that your manuscript meets PLOS ONE's style requirements, including those for file naming. The PLOS ONE style templates can be found at http://www.plosone.org/attachments/PLOSOne_formatting_sample_main_body.pdf and http://www.plosone.org/attachments/PLOSOne_formatting_sample_title_authors_affiliations.pdf | Thank you for your advice. We have downloaded these files and checked the format. |
| 2. Please include additional information regarding the survey or questionnaire used in the study and ensure that you have provided sufficient details that others could replicate the analyses. For instance, if you developed a questionnaire as part of this study and it is not under a copyright more restrictive than CC-BY, please include a copy, in both the original language and English, as Supporting Information. In addition, please ensure you include any details of pre-testing of this questionnaire, i.e. how many participants were involved and from where were they recruited. | We have uploaded two copies of the questionnaire, one in Chinese and the other one in English, as supporting files.  Pre-investigation was conducted among 20 HIV positive MSM in Hangzhou city to improve the quality of questionnaire, mainly in questions related to sexual contacts with their sexual partners. |
| 3. Please state what type of consent was obtained from guardians of minors included in this study, ensure that you have specified (1) whether consent was informed and (2) what type you obtained (for instance, written or verbal, and if verbal, how it was documented and witnessed). | Consent was informed and written informed consent forms were obtained from HIV positive minor participants and their guardians (lines 121-123). We make it clear in the method section. |
| 4. Thank you for stating the following in the Acknowledgments Section of your manuscript:  "Funding for the costs of publication in an open-access, peer-reviewed journal was supported by Key Project on Social Development among S&T Major Project of Zhejiang Province, China (2013C03047-1), Zhejiang Provincial Medicine Science and Technology Plan(2015PYA004), National Science and Technology Major Project of China (2017ZX10201101), The Training Project of Young Scientific and Technological Innovative Talents of Zhejiang Provincial Center of Disease Control and Prevention."  We note that you have provided funding information that is not currently declared in your Funding Statement. However, funding information should not appear in the Acknowledgments section or other areas of your manuscript. We will only publish funding information present in the Funding Statement section of the online submission form.  Please remove any funding-related text from the manuscript and let us know how you would like to update your Funding Statement. Currently, your Funding Statement reads as follows:  "The funders had no role in study design, data collection and analysis, decision to publish, or preparation of the manuscript." | Thanks. We have removed details of funding information from the manuscript to the online submission form.  The funding statement will be updated as follows:  “This study was supported by Key Project on Social Development among S&T Major Project of Zhejiang Province, China (2013C03047-1), Zhejiang Provincial Medicine Science and Technology Plan (2015PYA004), National Science and Technology Major Project of China (2017ZX10201101), The Training Project of Young Scientific and Technological Innovative Talents of Zhejiang Provincial Center of Disease Control and Prevention.  The funders had no role in study design, data collection and analysis, decision to publish, or preparation of the manuscript.” |
| 5. We note that you have indicated that data from this study are available upon request. PLOS only allows data to be available upon request if there are legal or ethical restrictions on sharing data publicly. For information on unacceptable data access restrictions, please see http://journals.plos.org/plosone/s/data-availability#loc-unacceptable-data-access-restrictions.  In your revised cover letter, please address the following prompts:  a) If there are ethical or legal restrictions on sharing a de-identified data set, please explain them in detail (e.g., data contain potentially identifying or sensitive patient information) and who has imposed them (e.g., an ethics committee). Please also provide contact information for a data access committee, ethics committee, or other institutional body to which data requests may be sent.  b) If there are no restrictions, please upload the minimal anonymized data set necessary to replicate your study findings as either Supporting Information files or to a stable, public repository and provide us with the relevant URLs, DOIs, or accession numbers. Please see http://www.bmj.com/content/340/bmj.c181.long for guidelines on how to de-identify and prepare clinical data for publication. For a list of acceptable repositories, please see http://journals.plos.org/plosone/s/data-availability#loc-recommended-repositories.  We will update your Data Availability statement on your behalf to reflect the information you provide. | We are sorry we couldn’t upload the database in the system because this database contains personal and sexual information of HIV positive persons, which is sensitive, confidential and under restrict management by Chinese Law. Please contact our ethics committee if having any question about this.  Contact information of Zhejiang Provincial Center for Disease Control and Prevention Ethics Review board:  Address: NO.3399, Binsheng Road, Binjiang District, Hangzhou City, Zhejiang Province, China.  Please contact: Mr. Zhenggang Jiang  Email: zhgjiang@cdc.zj.cn，TEL：+86 571-87115105 |
| 6. Your ethics statement must appear in the Methods section of your manuscript. If your ethics statement is written in any section besides the Methods, please move it to the Methods section and delete it from any other section. Please also ensure that your ethics statement is included in your manuscript, as the ethics section of your online submission will not be published alongside your manuscript. | We have checked and moved ethics statement to the methods section, and deleted ethics statement in any other sections. |
