# Supplementary material for: HIV testing uptake and yield among sexual partners of HIV-positive men who have sex with men in Zhejiang Province, China, 2014-2016: A cross-sectional pilot study of a choice-based partner tracing and testing package
Source: PLoS One. 2020 Jun 4;15(6):e0232268. doi: 10.1371/journal.pone.0232268 (PMC7272034; doi:10.1371/journal.pone.0232268)
Supplement: Attachment — Submitted filename: Response to Reviewers.docx [file pone.0232268.s008.docx]

**Response to Reviewers**

Dear editor and reviewers，

We appreciate a lot for your comments which are critical to help us improve this manuscript. We take these comments into account carefully, and have revised this manuscript accordingly. All authors of this manuscript would like to express our sincere thanks for your consideration of this resubmission.

The reviewers’ comments are responded as follows point by point.

Reviewer #2: The authors have addressed most points satisfactorily, but there are a few outstanding issues.

1. The authors state: “The Chinese government has advocated for an increase in HIV

testing among high-risk populations since 2012.” However, it is clear that testing among high-risk populations was advocated much earlier than 2012. See http://data.unaids.org/una-docs/china_joint_assessment_2003_en.pdf

- Thanks a lot for your comment. We have checked this assessment and related polices，and read the document *A Joint Assessment of HIV/AIDS Prevention, Treatment and Care in China* provide by the reviewer. In 2003, Chinese government issued the national policy of "Four Free and One Care", which included free HIV counselling and testing (VCT), free antiviral treatment, free prevention of transmission from mother to child, free education for HIV affected children, and care for people living with HIV. We have revised this sentence into “In 2003, the Chinese government began to enforce the free voluntary counselling and testing (VCT) policy.” In lines 51-53. The relevant reference was also changed.

2. Line 135. Change “HIV men” to men living with HIV, or something else.

- We have revised “HIV men” into “HIV positive MSM”, in lines 134.

3. Lines 142-145. “Enrolled participants proceeded to the next round if tested…” What do you mean by “the next round if tested”? Please restructure/reword this paragraph, which I found to be somewhat confusing.

- This sentence has been revised as “Enrolled sexual partners proceeded to the next round if tested HIV positive and consented to be enrolled as ICs.” In lines 141-142.

4. Line 234. “to identify a positive.” Please reword.

- We have revised the whole sentence into “Overall, the proportion of HIV testing among reachable sexual partner for HIV testing was 87%, and HIV testing yield among tested sexual partners was 16%.” In lines 231-233.

5. There are still typos/grammatical issues in the manuscript. For example, “sexual partner” on lines 243 and 245 and Table 2 (Frequency of sexual partner) should be changed to “sex”. And “mod” is misspelled on line 254.

- We have checked and revised these grammatical or spelling mistakes.

6. Lines 291-292. “The mean number of successfully recruiting a reachable

292 sexual partner for HIV testing was 1.1.” Please rephrase for clarity.

- We think this sentence may be a little redundant and not clear, and removed this sentence.

7. Lines 329-331. “This may reflect the fact that the choice of information assisted mode may indicate a less intimate relationship.” Please elaborate on the connection between an intimate relationship and lower effectiveness in case finding. That connection may not be intuitive to the reader.

- This sentence has been revised in lines 329-330 to clarify that less intimate relationship may result in the difficulty for ICs to reach their sexual partners.

8. Grammar. Line 350. “However, the gap between total disclosed sexual partners (4116) and

351 those in recent six months (1014) is also need to be considered, as…”

- This sentence has been revised as “However, the number of sexual partners in recent six months (1,014) was much less than the number of sexual partners in their whole life (4,116), which is also need to be considered, as…” in lines 349-350.

9. Line 358. Given the stated study population, I don’t quite understand the issue with selection bias. The stated study population on lines 103-106 is MSM who agreed to participate in the program. This would suggest that MSM who did not agree to participate were NOT in the study population of interest. Perhaps the authors meant the study sample were MSM who tested positive and agreed to participate and that the study population was all MSM who tested positive?

- As descripted in lines 102-105, we defined the study population as newly diagnosed HIV-positive MSM and their sexual partners, who agreed to participate in the partner tracing and HIV testing program at VCT clinic study sites in the cities of Hangzhou and Ningbo from June 2014 through June 2016. We agree that it may not be suitable to name this as selection bias. This sentence is revised into “The demographic characteristics of HIV positive MSM might be associate with their participation in this pilot study” 357-359.

10. The wording of the stated aim could be clearer. “In this study, we describe a pilot study of a choice-based partner tracing and testing program aimed at analyzing the uptake and infection status of HIV testing for sexual partners of newly diagnosed HIV-positive MSM.” Is it the study that is aiming to analyze the uptake and infection status? Or is it the program itself that aimed to analyze the uptake and infection status?

- This study aims to analyze the uptake and the infection status. We have revised this sentence into “In this study, we aimed at analyzing the uptake and infection status of HIV testing for sexual partners of newly diagnosed HIV-positive MSM.” In lines 95-97.
